# Effects of hydrogen-rich water in a rat model of polycystic kidney disease

**DOI:** 10.1371/journal.pone.0215766

**Published:** 2019-04-23

**Authors:** Masatora Yamasaki, Motoaki Miyazono, Maki Yoshihara, Atsuhiko Suenaga, Masato Mizuta, Makoto Fukuda, Shuichi Rikitake, Yuji Ikeda

**Affiliations:** Department of Nephrology, Faculty of Medicine, Saga University, Saga, Japan; Max Delbruck Centrum fur Molekulare Medizin Berlin Buch, GERMANY

## Abstract

Various factors are considered to be mechanisms of the increase in the sizes of cysts in patients with polycystic kidney disease. Vasopressin is one of the causes, and drinking large volumes of water shows an effect of suppressing an increase in cysts. On the other hand, it is known that hydrogen-rich water reduces oxidative stress and has a good effect on kidney injury. We examined whether drinking large volumes of hydrogen-rich water affected the increase in the sizes of cysts. Forty 5-week-old *PCK* rats were randomly assigned to four groups: C(Control), purified water; W(Water), water with sugar; H(Hydrogen), hydrogen-rich water; WH(Water+Hydrogen), hydrogen-rich water with sugar. They consumed water from 5 to 15 weeks of age. The intake of water in the groups in which sugar was added to the water (W, WH) significantly increased in comparison to C, but there was no significant change in the serum Creatinine concentration. The kidney weight per body weight in W was significantly decreased in comparison to C. The kidney weights in H and WH were significantly increased in comparison to W. There were no significant differences in the ratio of the cross-sectional area of the cysts to the whole area among the groups. This experiment showed that the effect of drinking large volumes of hydrogen-rich water was not significantly different from that of normal water, in terms of preventing an increase in the size of cysts in *PCK* rats. However, some papers acknowledge the influence of hydrogen water. Significant differences might become obvious if we change aspects such as the administration method or administration period.

## Introduction

Autosomal-dominant polycystic kidney disease (ADPKD) causes multiple cysts in the bilateral kidneys, which increases and impairs normal kidney tissue. Approximately half ADPKD patients progress to end-stage renal failure by 60 years of age. ADPKD is the most common hereditary kidney disease, and affects one 1 in 4,000 people in Japan, and is the cause of dialysis in 3.5% of dialysis patients [1]. There have been no specific treatments until recent years. However, tolvaptan, a vasopressin V2-receptor antagonist, was reported to have inhibitory effects against the increase in kidney volume and the reduction in the kidney function [2].

Tolvaptan is now used in various countries to suppress the progression of ADPKD because vasopressin is considered to be a biologically exacerbating factor in the tubular cells of ADPKD and cyst formation is thought to be suppressed in a state of vasopressin deficiency [3]. The dilution power of urine is reported to be kept when the kidney function is in the normal range. It is thought that it is possible to suppress vasopressin by merely increasing the water load if there are no problems with a patient’s cardiac or kidney functions [4]. Thus, the usefulness of drinking large volumes of water in daily life was considered, and it was reported that drinking large volumes of water suppressed cyst development in a rat model of ADPKD [5]. On the other hand, it is known that hydrogen-rich water reduces oxidative stress and has a good effect on kidney injury [6–7]. Regarding the relationship between oxidative stress and the progression of ADPKD, a study using PKD model mice/rats demonstrated an increase in oxidative stress marker levels and the decreased expression of antioxidant enzymes [8]. The oxidative stress marker levels of ADPKD patients have been reported to be higher than those of healthy subjects [9]. It is also reported that vascular oxidative stress and inflammation develop with ADPKD [10]. Thus, we hypothesized that drinking large volumes of hydrogen-rich water would alleviate oxidative stress as well as suppress vasopressin, which may contribute to inhibit the development of cystic kidney and suppress the progression of renal disorder. In *PCK* rats, cysts form in the collecting duct due to a mutation in the pkhd1 gene. This gene is related to the gene responsible for human autosomal-recessive polycystic kidney disease (ARPKD) and follows a similar clinical process to ADPKD. *PCK* rats are therefore used as models of ADPKD [11]In this study, we investigated the effect of the intake of large volumes of hydrogen-rich water in *PCK* rats.

## Materials and methods

### Ethics statement

All experiments were approved by the Animal Experimental Ethical Committee of Saga University (Permit Number: 28-065-0), and were conducted in compliance with our institutional guidelines and with international standards for the manipulation and care of laboratory animals. All surgical operations were performed under isoflurane anesthesia, and all efforts were made to minimize suffering.

### Animals

Forty *PCK* rats, a rat model of PKD, were used for this experiment. The *PCK* rat strain used in the current study was originally derived from a Sprague-Dawley colony from Charles River, Japan. The descendants were bred and maintained at the Education and Research Center of Animal Models for Human Diseases, Fujita Health University. *PCK* rats form cysts in the collecting duct due to a mutation of the pkhd1 gene. This gene related to the gene responsible for human autosomal-recessive polycystic kidney disease (ARPKD), but follows a similar clinical process to ADPKD. PCK rats develop progressive cystic enlargement of the kidneys from one week of age. The renal cysts developed as a focal process from thick ascending loops of Henle, distal tubules, and collecting ducts in the corticomedullary region and outer medulla [11].Thus, *PCK* rats are used as a model of ADPKD.

### Methods

Forty 5-week-old *PCK* rats were randomly divided into 4 groups. C (Control), purified water; W (Water), water with sugar; H (Hydrogen), hydrogen-rich water; G4, hydrogen-rich water with sugar. The water was consumed from 5 to 15 weeks of age. Sugar was added to the water to promote drinking. We used 5% glucose based on previous studies [5]. Hydrogen-rich water (1.6 ppm) was prepared using Aquela blue (Ecomo International, Fukuoka, Japan). For hydrogen-rich water, sugar hydrogen-rich water, an aluminum pouch was used as a container to prevent hydrogen volatilization. The rats were kept in standard cages under a 12-h light/dark cycle. The urine volume and amount of water consumed over 24 hours were measured at 15 weeks. At 15 weeks, the rats were anesthetized with isoflurane. Then both kidneys were isolated and the rats were exsanguinated. Blood sampling was performed at the same time. The total kidney weight was measured. The right kidney was fixed in 10% formalin, and HE-stained paraffin sections were prepared. The sections were photographed with a CCD camera to examine the size of the cyst. The images were analyzed using the ImageJ software program (National Institutes of Health, Maryland, United States of America) to calculate the ratio of cysts to the whole area.

### Statistical analysis

The data are shown as the median and interquartile range. Because some data did not show equal variance, a nonparametric test was adopted. We performed the Steel Dwass test for multiple comparisons of all pairs. All statistical analyses were performed using the JMP Pro 12 software program (SAS Institute Japan, Tokyo, Japan).

## Results

### Drinking water volume and urine volume

**Fig 1:**
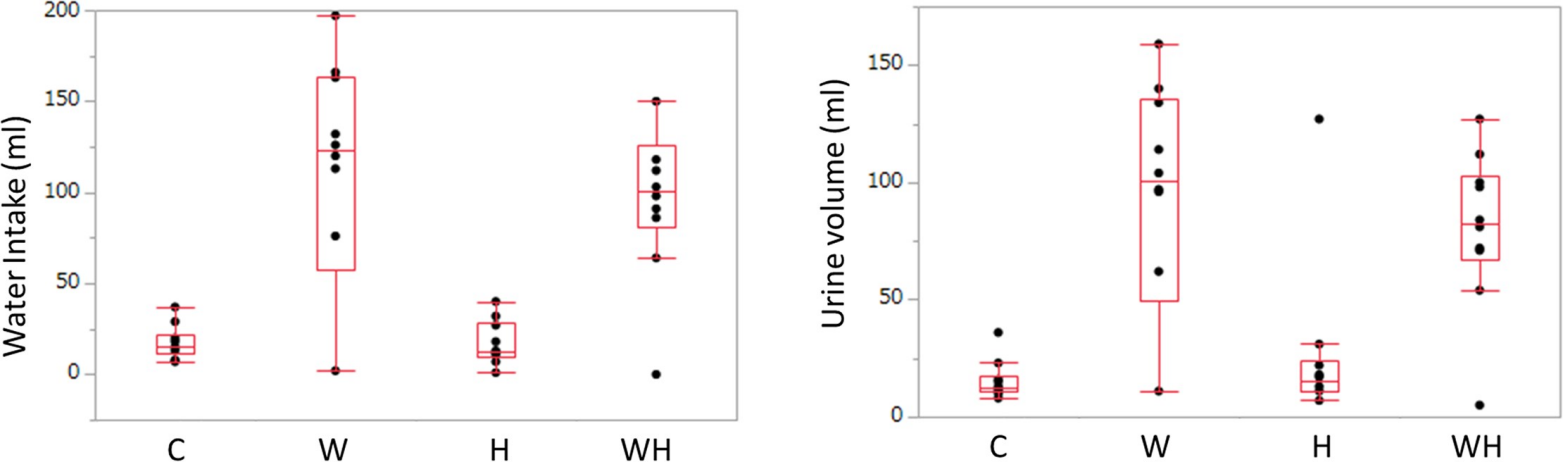
The volume of water intake and the urine volume. (A) The volume of water intake (ml). Significant differences (P<0.05) were observed between C and WH (p<0.0147), and H and WH (p = 0.0149). (B) The urine volume at 15 weeks (ml). Significant differences (P<0.05) were observed between C and W (p = 0.0445), C and WH (p = 0.0150), and W and H (p = 0.0011).

The drinking water volumes were as follows: C, 15.00 (11.75–22.25) ml; W, 123.00 (57.50–163.75) ml; H, 12.5 (10.00–28.25) ml; and WH, 100.50 (80.50–126.00) ml. In comparison to C, the volume of water was significantly increased in WH (p = 0.0147). Furthermore, the volume of drinking water consumed in WH was significantly greater than that in H (p = 0.0149) (Fig 1A). The urine volumes of the groups were as follows: C, 12.50 (10.75–17.75) ml; W, 100.50 (49.25–135.50) ml; H, 15.00 (11.00–24.25) ml; and WH, 82.50 (66.75–103.00) ml. In comparison to C, the urine volume was significantly increased in the groups to which sugar was added (W, WH). The urine volume of H did not differ from that of C to a statistically significant extent (W: p = 0.0445, WH: p = 0.0150) (Fig 1B).

### BUN and Cr

**Fig 2:**
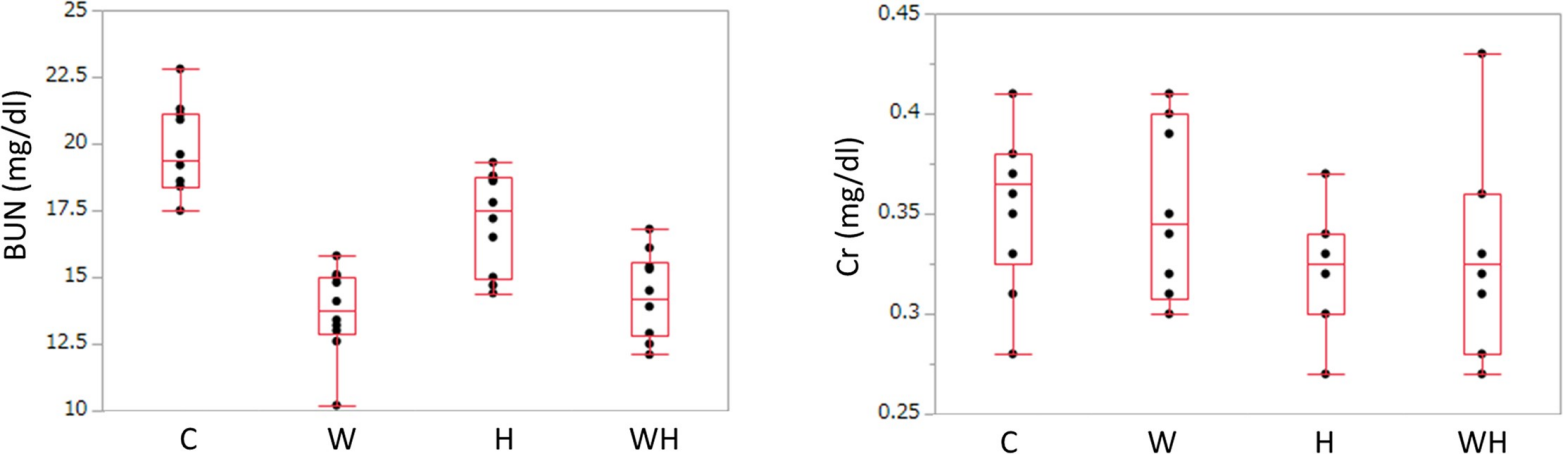
The BUN concentrations and the serum concentrations. (A) The BUN concentrations at 15 weeks (mg/dl). Significant differences (P<0.05) were observed between C and W (p = 0.0010), C and WH (p = 0.0010), W and H (p = 0.0168), and H and WH (p = 0.0365). (B) The serum Cr concentrations at 15 weeks (mg/dl). No significant differences (p<0.05) were observed between any of the groups.

Markers of the renal function were assessed. The blood urine nitrogen (BUN) levels at 15 weeks were as follows: C, 19.40 (18.40–21.15) mg/dl; W: 13.75 (12.9–15.02) mg/dl; H, 17.50 (14.92–18.72) mg/dl; and WH, 14.20 (12.80–15.57) mg/dl there were. In comparison to C, the BUN levels were significantly lower in the groups with added sugar (W, p = 0.0010; WH, p = 0.0010). Furthermore, the BUN levels of W and WH were significantly lower in comparison to H (W, p = 0.0168; WH: p = 0.0365). However, there was no significant difference between W and WH (p = 0.9418) (Fig 2A). The creatinine (Cr) levels of the four groups were as follows: C, 0.365 (0.325–0.380) mg/dl; W, 0.345 (0.307–0.400) mg/dl; H, 0.325 (0.300–0.340) mg/dl; and WH, 0.325 (0.280–0.360) mg/dl. There were no significant differences among the groups (Fig 2B). We measured the creatinine clearance and performed the Steel Dwass test in the same way, but no significant difference was noted. (S1 Table)

### The kidney weight to body weight ratio

**Fig 3:**
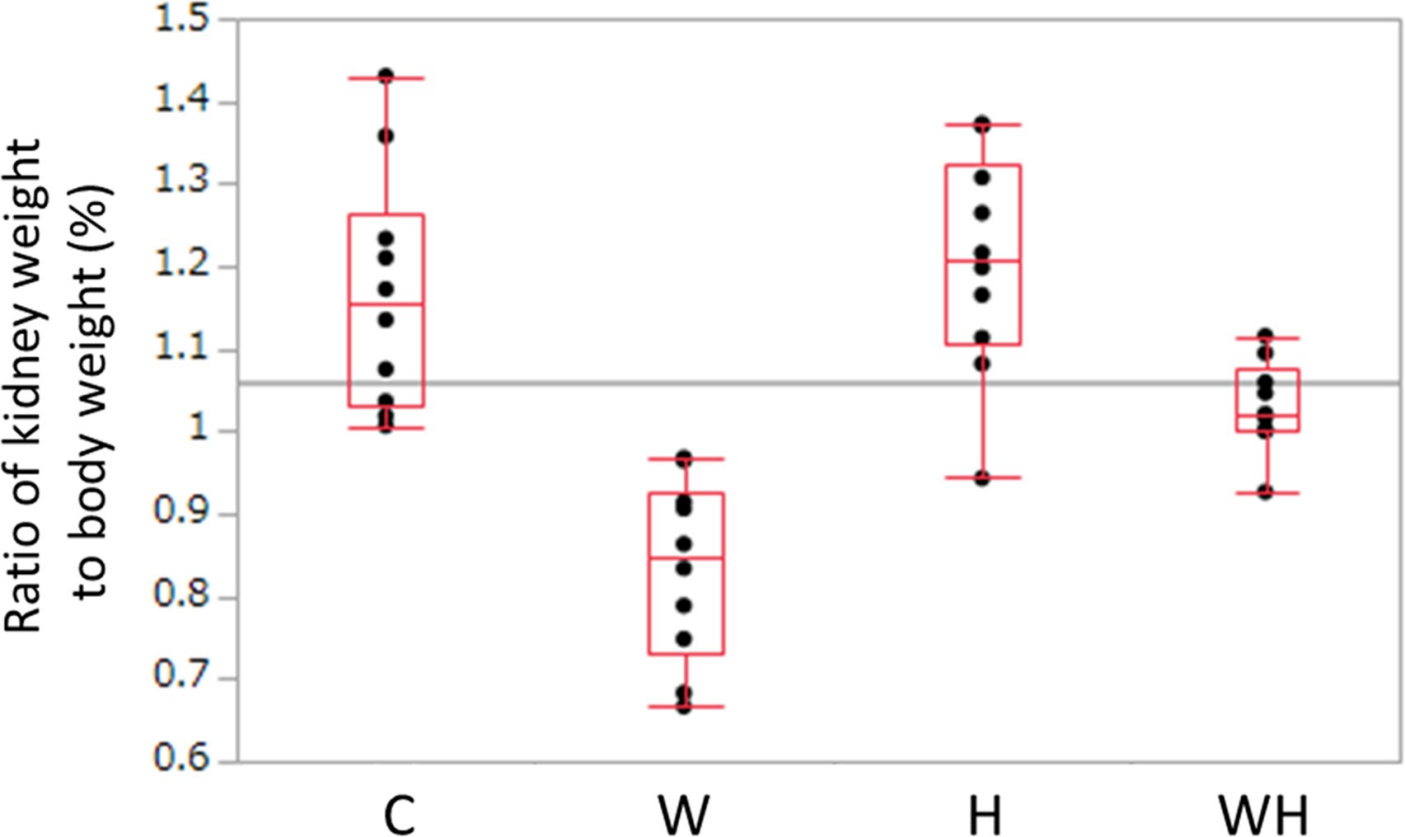
The ratio of kidney weight to body weight. The ratio of kidney weight to body weight at 15 weeks (%). Significant differences (P<0.05) were observed between C and W (p = 0.0010), W and H (p = 0.0019), W and WH (p = 0.0029), and H and WH (p = 0.0317).

The kidney weight to body weight ratios were as follows: C, 1.153% (1.031–1.264); W, 0.848% (0.732–0.926); H, 1.207% (1.105–1.323); and WH, 1.021% (1.000–1.077) (Fig 3). In comparison to C, W was significantly lighter (p = 0.0010). The ratios of H and WH did not differ from the ratio of C to a statistically significant extent (H: p = 0.889, WH: p = 0.111). The ratios of H and WH were significantly higher than the ratio of W (H: p = 0.0019, WH: p = 0.0029). It was not possible to measure the kidney weight of one rat in WH to a measurement mistake.

### The ratio of the cystic area to the in the cross-section of the right kidney

**Fig 4:**
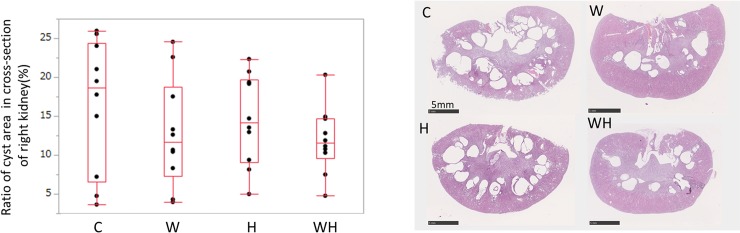
The cross-section of the right kidney. (A) The ratio of the area of cysts in the cross-section of the right kidney at 15 weeks (%). No significant differences (p<0.05) were observed between any of the groups. (B) A typical cross-section of right kidney in each group.

The ratio of the cystic area to the in the cross-section of the right kidney in the four groups were as follows: C, 18.62% (6.59–24.4); W, 11.64% (7.30–18.78); H, 14.11% (9.06–19.69); and WH, 11.51% (9.59–14.72) (Fig 4A and 4B). The ratio was not significantly different between all the groups.

### Other data

We measured the level of urine protein as a marker for evaluating the severity of PKD and the expression of 8-OHdG as a marker of oxidative stress using all residual urine. However, nearly half of the values in both data sets were below the measurement sensitivity, so the evaluation of the urinary protein level was thought to be difficult (S1 Table).

## Discussion

ADPKD is caused by mutations of two genes (PKD1, PKD2) encoding Polycystin 1 (PC 1) and Polycystin 2 (PC 2). Thus far, there is no fundamental therapy for the disease. In patients with an increased kidney volume, the renal function gradually declines, progressing to renal failure, and renal replacement therapy is required. In polycystic kidney (PKD) cells, the functional abnormality reduces the concentration of intracellular Ca^2+^, the activity of phosphodiesterase (PDE) enzyme, which degrades cyclic AMP (cAMP), decreases and the intracellular cAMP concentration increases. As a result, the function of cAMP-dependent protein kinase A (PKA) is enhanced, various signal pathways (EGF/EGFR, Wnt, Raf/MEK/ERK, JAK/STAT, mTOR, and others) are activated, and cell proliferation occurs. On the other hand, the vasopressin (AVP) R2 receptor present in the renal tubule (collecting duct) is activated by AVP and works to increase water permeability through adnylcyclase (AC) and cAMP to retain moisture. In PKD cells, the increase in cAMP caused by vasopressin increases the sizes of cysts. Thus, drinking a large amount of water and tolvaptan (a vasopressin V 2 receptor) antagonist is expected to suppress the increase in cyst numbers and suppress the progression of renal disorder [12].

Hydrogen in aqueous solution shows antioxidant action by reducing hydroxyl radical. Various antioxidant treatments against oxidative stress, such as valdoxoron methyl and N-acetyl cysteine, are attracting attention in kidney injury research [13–14]. Li FY et al. reported that renal damage and carcinogenesis were suppressed in rats by the intraperitoneal administration of the carcinogen, trisodium nitrilotriacetate, which were then allowed *ad libitum* access to hydrogen-rich water [6]. Xin HG et al. reported that the consumption of hydrogen-rich water alleviated renal injury in spontaneous hypertensive rats [7].

Treatment with tolvaptan also requires a large amount of drinking water to compensate for the dehydration caused by water diuresis. Thus, this study was conceived to investigate whether a synergistic action with the antioxidant effect could be achieved using hydrogen-rich water as drinking water. The effect of inhibiting cyst growth (as assessed by kidney weight) and the effect of suppressing the renal deterioration function (as assessed by the creatinine level) were not demonstrated.

Hydrogen dissolves in water at up to 1.6 ppm under room atmospheric pressure. The dissolved hydrogen concentration varies greatly depending on the machine used in its manufacture, subsequent storage containers and conditions, and other factors. The difference may have a significant influence on the efficacy of hydrogen-rich water. Xue et al. experimented with aluminum bags [15]. Based on their experiments, we used aluminum bags in the same way, by devising a drinking spout, we took sufficient care to prevent the hydrogen from volatilizing. However, it was drinking water to which the animals had *ad libitum* access; thus, the problem of stability such regarding the amount of hydrogen remained.

Hydrogen is also thought to increase the ATP production in mitochondria [16]. ATP is converted intracellularly into cAMP by AC. Thus, as cAMP—which is involved in the increase in the cysts—increases, the antioxidant effect may be canceled out. The observation period of the present study was 10 weeks (from 5 weeks to 15 weeks of age), and it is possible that hydrogen-rich water would have had a more significant effect over a longer observation period.

At any rate, hydrogen-rich water can be produced with relatively little effort, while taking tolvaptan requires the intake of large amounts of water. If its effectiveness can be improved by changing the water to hydrogen-rich water, it might be a useful treatment with few side effects. It is suggested that hydrogen-rich water is useful not only for kidney injury but also for diseases of various organs [15, 17–18], and we believe that its usefulness is worth investigating further.

## Conclusion

In our trial, there was no significant difference in the creatinine level or cyst size. However, some papers acknowledge the influence of hydrogen water. Significant differences might become obvious if we change aspects such as the administration method or administration period.

## Supporting information

S1 Table Urine data. (DOCX)

S2 Table Other data. (DOCX)
