# Supplementary material for: Effects of hydrogen-rich water in a rat model of polycystic kidney disease
Source: PLoS One. 2019 Apr 23;14(4):e0215766. doi: 10.1371/journal.pone.0215766 (PMC6478309; doi:10.1371/journal.pone.0215766)
Supplement: S1 Table — (DOCX) [file pone.0215766.s001.docx]

| **Table1 Urine data** | | | | | | | | | |
| --- | --- | --- | --- | --- | --- | --- | --- | --- | --- |
| Group | No. | Urine Protein (mg/dL) | Urine 8-OHDG  (ng/mL) | Urine Cr (mg/dL) | Urine volume  (mL) | Serum Cr  (mg/dL) | Daily urine protein  (mg) | Daily urine 8-OHDG　 (ng) | CrCl  (mL/min) |
| C | 1 | 6.1 | 8.55 | 125.94 | 16 | 0.31 | 0.98 | 136.75 | 4.51 |
| C | 2 | 3.9 | 7.56 | 99.84 | 23 | 0.33 | 0.90 | 173.80 | 4.83 |
| C | 3 | <LOD | <LOD | 112.72 | 36 | 0.38 | <LOD | <LOD | 7.42 |
| C | 4 | <LOD | 14.37 | 183.82 | 10 | 0.35 | <LOD | 143.74 | 3.65 |
| C | 5 | <LOD | 3.37 | 112.12 | 11 | 0.36 | <LOD | 37.06 | 2.38 |
| C | 6 | 3.5 | <LOD | 17.92 | 12 | 0.41 | 0.42 | <LOD | 0.36 |
| C | 7 | 3.1 | 11.29 | 10.67 | 15 | 0.37 | 0.47 | 169.41 | 0.30 |
| C | 8 | 2.2 | 14.38 | 15.14 | 13 | 0.28 | 0.29 | 187.00 | 0.49 |
| C | 9 | 4 | <LOD | 15.04 | 8 | 0.37 | 0.32 | <LOD | 0.23 |
| C | 10 | 3.4 | <LOD | 141.03 | 12 | 0.38 | 0.41 | <LOD | 3.09 |
| W | 1 | 7.8 | <LOD | 71.23 | 140 | 0.35 | 10.92 | <LOD | 19.79 |
| W | 2 | 7.1 | <LOD | 116.70 | 11 | 0.40 | 0.78 | <LOD | 2.23 |
| W | 3 | 4 | 10.72 | 54.98 | 62 | 0.31 | 2.48 | 664.50 | 7.64 |
| W | 4 | <LOD | 13.11 | 178.78 | 134 | 0.32 | <LOD | 1757.25 | 51.99 |
| W | 5 | <LOD | 3.13 | 94.26 | 97 | 0.30 | <LOD | 304.07 | 21.16 |
| W | 6 | <LOD | <LOD | 21.88 | 96 | 0.41 | <LOD | <LOD | 3.56 |
| W | 7 | 2.4 | <LOD | 15.59 | 159 | 0.39 | 3.82 | <LOD | 4.41 |
| W | 8 | 2.2 | <LOD | 263.40 | 114 | 0.30 | 2.51 | <LOD | 69.51 |
| W | 9 | 4.9 | <LOD | 32.47 | 104 | 0.40 | 5.10 | <LOD | 5.86 |
| W | 10 | 2.5 | 15.82 | 17.51 | 11 | 0.34 | 0.28 | 173.97 | 0.39 |
| H | 1 | 3.9 | 1.86 | 90.20 | 127 | 0.30 | 4.95 | 235.96 | 26.52 |
| H | 2 | 7.5 | <LOD | 72.77 | 11 | 0.30 | 0.83 | <LOD | 1.85 |
| H | 3 | 2 | 0.41 | 49.45 | 18 | 0.27 | 0.36 | 7.41 | 2.29 |
| H | 4 | <LOD | 5.44 | 164.29 | 13 | 0.34 | <LOD | 70.66 | 4.36 |
| H | 5 | <LOD | 9.63 | 126.89 | 11 | 0.34 | <LOD | 105.90 | 2.85 |
| H | 6 | 5.5 | 9.48 | 13.55 | 22 | 0.37 | 1.21 | 208.57 | 0.56 |
| H | 7 | <LOD | 2.66 | 127.11 | 13 | 0.32 | <LOD | 34.61 | 3.59 |
| H | 8 | 6.6 | <LOD | 29.41 | 31 | 0.33 | 2.05 | <LOD | 1.92 |
| H | 9 | 2.2 | <LOD | 13.49 | 7 | 0.32 | 0.15 | <LOD | 0.20 |
| H | 10 | 4.5 | <LOD | 18.92 | 17 | 0.34 | 0.77 | <LOD | 0.66 |
| WH | 1 | 2.4 | 8.63 | 48.11 | 127 | 0.32 | 3.05 | 1095.58 | 13.26 |
| WH | 2 | <LOD | 13.56 | 135.84 | 72 | 0.27 | <LOD | 976.41 | 25.16 |
| WH | 3 | <LOD | 3.06 | 83.36 | 84 | 0.28 | <LOD | 256.67 | 17.37 |
| WH | 4 | <LOD | <LOD | 109.47 | 100 | 0.31 | <LOD | <LOD | 24.52 |
| WH | 5 | <LOD | <LOD | 121.48 | 71 | 0.28 | <LOD | <LOD | 21.39 |
| WH | 6 | 5.2 | <LOD | 12.29 | 81 | 0.33 | 4.21 | <LOD | 2.09 |
| WH | 7 | 6.5 | 10.64 | 22.32 | 112 | 0.43 | 7.28 | 1191.40 | 4.04 |
| WH | 8 | 5.6 | 5.67 | 20.23 | 5 | 0.36 | 0.28 | 28.33 | 0.20 |
| WH | 9 | 5 | 9.94 | 16.95 | 54 | 0.36 | 2.70 | 536.83 | 1.77 |
| WH | 10 | <LOD | <LOD | 22.58 | 98 | 0.33 | <LOD | <LOD | 4.66 |
| <LOD: below the limit of detection | | | | | | | | | |
