# Supplementary material for: Effects of hydrogen-rich water in a rat model of polycystic kidney disease
Source: PLoS One. 2019 Apr 23;14(4):e0215766. doi: 10.1371/journal.pone.0215766 (PMC6478309; doi:10.1371/journal.pone.0215766)
Supplement: S2 Table — (DOCX) [file pone.0215766.s002.docx]

| **Table 2 other data** | | | | | | |
| --- | --- | --- | --- | --- | --- | --- |
| Group | No. | drinking volume(mL) | BUN(mg/dL) | body weight(g) | kidney weight(g) | The ratio of cysts area(%) |
| C | 1 | 18 | 18.4 | 477 | 4.86 | 4.746835443 |
| C | 2 | 29 | 18.4 | 518 | 5.57 | 7.207207207 |
| C | 3 | 37 | 18.6 | 450 | 6.11 | 15 |
| C | 4 | 7 | 19.6 | 504 | 6.1 | 25.55205047 |
| C | 5 | 15 | 20.9 | 505 | 5.92 | 3.636363636 |
| C | 6 | 13 | 19.2 | 468 | 4.85 | 17.76315789 |
| C | 7 | 15 | 22.8 | 460 | 6.58 | 21.03658537 |
| C | 8 | 20 | 17.5 | 454 | 5.6 | 19.49458484 |
| C | 9 | 15 | 21.1 | 459 | 5.21 | 24.02234637 |
| C | 10 | 8 | 21.3 | 438 | 4.41 | 25.95870206 |
| W | 1 | 163 | 15 | 523 | 4.74 | 8.309455587 |
| W | 2 | 2 | 15.8 | 509 | 4.91 | 13.29479769 |
| W | 3 | 76 | 13.4 | 537 | 4.91 | 12.61829653 |
| W | 4 | 166 | 14.1 | 530 | 4.42 | 3.96039604 |
| W | 5 | 120 | 14.8 | 528 | 5.11 | 24.5398773 |
| W | 6 | 113 | 15.1 | 464 | 3.17 | 22.58064516 |
| W | 7 | 197 | 13 | 457 | 3.42 | 10.42253521 |
| W | 8 | 132 | 10.2 | 477 | 3.18 | 10.66666667 |
| W | 9 | 126 | 12.6 | 520 | 4.49 | 17.52265861 |
| W | 10 | 2 | 13.2 | 502 | 3.96 | 4.296875 |
| H | 1 | 40 | 18.7 | 475 | 4.48 | 8.139534884 |
| H | 2 | 12 | 16.5 | 478 | 5.57 | 20.7253886 |
| H | 3 | 13 | 18.8 | 480 | 6.07 | 19.15492958 |
| H | 4 | 12 | 18.6 | 464 | 5.02 | 4.98687664 |
| H | 5 | 11 | 17.8 | 444 | 5.4 | 22.32704403 |
| H | 6 | 27 | 17.2 | 448 | 6.15 | 9.375 |
| H | 7 | 7 | 19.3 | 457 | 6.26 | 13.53135314 |
| H | 8 | 32 | 14.7 | 484 | 5.8 | 19.35483871 |
| H | 9 | 1 | 14.4 | 478 | 6.25 | 14.69816273 |
| H | 10 | 18 | 15 | 476 | 5.3 | 12.9476584 |
| WH | 1 | 150 | 13.9 | 450 | 4.17 | 4.761904762 |
| WH | 2 | 91 | 16.1 | 496 | 5.19 | 7.491856678 |
| WH | 3 | 103 | 15.3 | 512 | 5.23 | 11.14982578 |
| WH | 4 | 112 | 13.9 | 517 | 5.66 | 12.8125 |
| WH | 5 | 86 | 14.5 | 509 | 5.1 | 11.875 |
| WH | 6 | 98 | 12.1 | 470 | 4.98 | 20.30848329 |
| WH | 7 | 150 | 16.8 | 453 | 4.53 | 10.29411765 |
| WH | 8 | 0 | 15.4 | 473 | 4.81 | 14.92063492 |
| WH | 9 | 64 | 12.5 | 476 | 5.31 | 10.77283372 |
| WH | 10 | 118 | 12.9 | 464 | missed | 14.66165414 |
|  | | | | | | |
